# Death Due to Intra-aortic Migration of Kirschner Wire From the Clavicle: A Case Report and Review of the Literature

**DOI:** 10.1097/MD.0000000000003741

**Published:** 2016-05-27

**Authors:** Lei Tan, Da-Hui Sun, Tiecheng Yu, Linxiang Wang, Dong Zhu, Yan-Hui Li

**Affiliations:** From the Departments of Orthopedic Trauma (LT, DH-S, TC-Y, LX-W, DZ); and Cardiology and Echocardiography (YH-L), The First Hospital of Jilin University, Changchun, China.

## Abstract

Migration of orthopedic fixation wires into the ascending aorta though a rare occurrence can have devastating consequences. Therefore, prompt recognition, with immediate and cautious retrieval of the implant is paramount in averting these complications.

We present a case of a 5-year-old boy with the intra-aortic migration of a K-wire used for the treatment of a right clavicle fracture. He was transferred to us with a history of syncope, chest pain, and shortness of breath 7 days after K-wire placement, which was performed at another hospital. On CT scan, the wire was found to be partially inside the ascending aorta, which was associated with massive hemopericardium and cardiac tamponade. The patient was taken up for emergency surgery for the removal K-wire and for the management of cardiac temponade. However, the patient developed cardiac arrest during the induction of intravenous anesthesia and endotracheal intubation. The K-wire was retrieved from the thorax via thoracotomy. However, the patient died 10 days after the surgery.

As the migration of wires and pins during orthopedic surgery can cause potentially fatal complications, these should be used very cautiously, especially for percutaneous treatment of shoulder girdle fractures. The patients with such implants should be followed frequently, both clinically and radiographically. If migration occurs, the patient should be closely monitored for emergent complications and the K-wire should be extracted immediately.

## INTRODUCTION

The use of metal pins and wires in orthopedic surgery was introduced in the early 20th century and was developed by Martin Kirschner. Kirschner wires (K-wire) are one of the earliest modalities for internal fixation of fractures in orthopedic surgery, especially in the management of fractures and dislocations of the shoulder. However, the migration of K-wires is not infrequent. The first case was reported in 1943^1^ and since then there have been 89 articles, which have reported 102 separate cases of K-wire migration. There are a few reports of postoperative pin migration from the shoulder girdle to the ascending aorta.^6–8,16,22–25,30,39,50^ Though uncommon, it is invariably associated with devastating consequences. Thus, prompt recognition, with immediate and cautious retrieval of the implant, is paramount in averting these complications. In this study, we present a case of a 5-year-old boy in whom a smooth K-wire, which was previously placed for fixation of right clavicle mortality fracture, migrated into the ascending aorta.

## CASE REPORT

**FIGURE 1:**
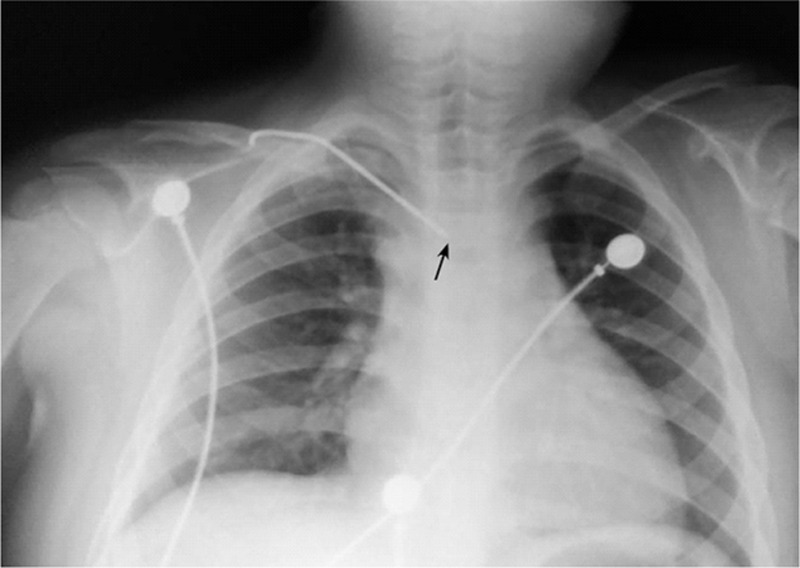
Chest x-ray showed migration of a bent Kirschner wire from the right clavicle. It was 9 cm in length, with its tip in the mediastinal shadow (black arrow).

**FIGURE 2:**
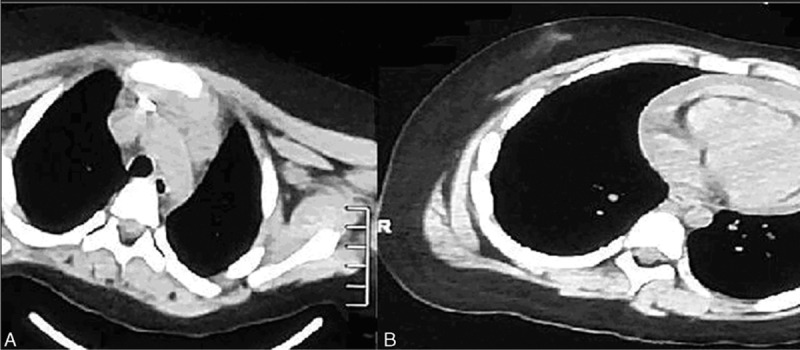
(A) CT scan showing that the migrated Kirschner wire was near the wall of ascending aorta. (B) CT scan showing massive hemopericardium and pericardial tamponade. CT scan = computed tomographic scan.

**FIGURE 3:**
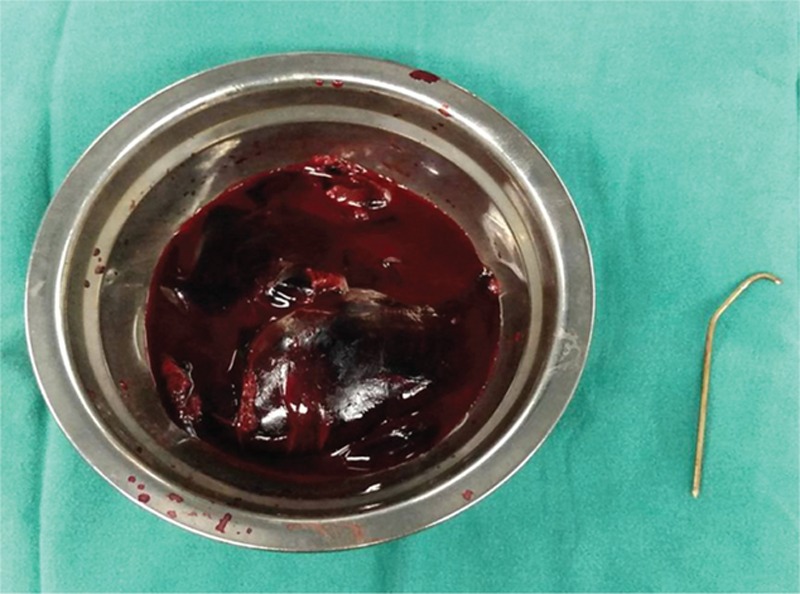
Intraoperative picture of dark red colored blood clot and the removed Kirschner wire.

The institutional review board (The First Hospital of Jilin University) approved this work and the informed consent was obtained. A 5-year-old boy was transferred to us with history of syncope, chest pain, and shortness of breath. The patient had a history of recent right midshaft clavicle fracture, sustained during a motorcycle accident 7 days earlier, for which he underwent open reduction and internal fixation with K-wires at another hospital. Physical assessment showed a blood pressure of 135/40 mm Hg, a pulse of 170 min^–1^, respiratory rate of 25 min^–1^. The radiographic examination revealed migration of the K-wire posteriorly into the mediastinum from the right clavicle (Figure 1). There was no pneumothorax, hemothorax, or pneumomediastinum. As injury to the great vessels was suspected, a computed tomographic (CT) scan was performed (Figure 2), which showed that the wire was partially inside the ascending aorta, leading to a massive hemopericardium and pericardial tamponade. The patient was immediately taken up for surgery for the removal K-wire and the management of cardiac tamponade. However, the patient developed cardiac arrest during the induction of intravenous anesthesia and endotracheal intubation. We performed closed cardiac massage immediately. At the same time, a median sternotomy was performed. The pericardium was found to be purple, and the pericardial cavity was filled with a dark red colored blood clot. Pericardiotomy was performed for the removal of the blood clot (Figure 3). While removing the blood clot, the patient developed ventricular fibrillation. The sinus rhythm was restored with cardiac compression and defibrillation. The electrocardiograph (ECG) showed significant S-T segment depression. Cardiopulmonary bypass was performed to assist cardiac function. After meticulous dissection, the K-wire was found to be piercing the ascending aorta near the anterior parietal pleura. However, the other great vessels and the trachea were not involved. The wire was carefully removed. The defect in the ascending aorta was repaired by direct suture.

The patient was in coma after the surgery and was transferred to the intensive care unit. Subsequently, the patient developed liver and kidney failure, and died 10 days after surgery.

## DISCUSSION

Migration is a rare but recognized complication of K-wire insertion. The incidence of iatrogenic injury due to K-wire migration is believed to be underestimated due to fear of subsequent litigation. Such complications, if not lethal, are associated with increased morbidity, often requiring surgical intervention. On reviewing articles from databases including PubMed, Elsevier, Cochrane Library, EMBASE, and Web of Science from the year 1943 to 2015, we found 102 cases of orthopedic wire migration.^2–13^ Majority of the cases of K-wire migration originated from the region of the shoulder girdle, including the proximal humerus,^14^ clavicle,^15^ the acromioclavicular joint, and sternoclavicular joints.^10,16,17^ However, a few cases originated from the finger,^18^ pelvis,^19,20^ hip,^21^ and rib.^48^ The final resting position of the orthopedic wire involved vascular structures (ascending aorta,^8,22–24^ pulmonary artery,^25,26^ and the heart^9,17,27–31^), lung,^32^ mediastinum,^33^ esophagus,^6^ spinal column,^5,10,12^ spleen,^34^ and the posthepatic retroperitoneal space.^35^ With respect to the distance of migration, long-distance migration of K-wires have been reported from the hip to the knee,^3^ the finger to the heart,^18^ the pelvis to the abdomen,^19^ the pelvis to the heart,^20^ and the hip to the liver.^21^ The short-distance migration into the chest may occur, such as when a wire placed in the shoulder girdle moves to the anterior mediastinum.^33^ The time period between implantation and detection of the migrated orthopedic wire varied widely. Fifty-six percent of the wires that migrated were noted within 3 months, whereas 74% were detected within 8 months of implant.^36^ Detection of migration within hours of implant has also been reported,^12,37^ whereas there have been reports where migrated wire was detected several years after placement.^8,27,28^

We reviewed the literature on iatrogenic aortic injuries due to K-wire migration. Fifteen patients were reported to have intra-aortic migration of K-wires.^7,8,14,16,17,22,29,23–25,30,38,39,50^ Most of the patients had cardiac tamponade, which was the cause of death in 4 cases.^14,16,29^ Three patients had pulmonary artery injury.^38,8,25^ Thirteen wires were lodged in the thoracic aorta, whereas 1 was lodged in the abdominal aorta.^50^ Thirteen patients subsequently underwent surgical removal of the wires by thoracotomy.

Our literature search found 11 deaths from catastrophic cardiovascular events caused by the migration of orthopedic wire following humeral fracture osteosynthesis (Table 1)^9,14^ and stabilization of sternoclavicular dislocation. The wire migrated to the ascending aorta (4 pins),^14,16,31,40^ the pulmonary artery (3 pins),^15,29^ the heart (3),^33,41,42^ and the lung (1 pin).^32^ Seven patients died from cardiac tamponade, 2 patients died from multiorgan failure^15,33^ and 1 patient died from irreversible ventricular fibrillation.^31^ In 3 cases, the wires were removed.^16,31,33^ Nine patients died within 3 months of surgery.^14,16,29,31,32,41,42^

**TABLE 1:**
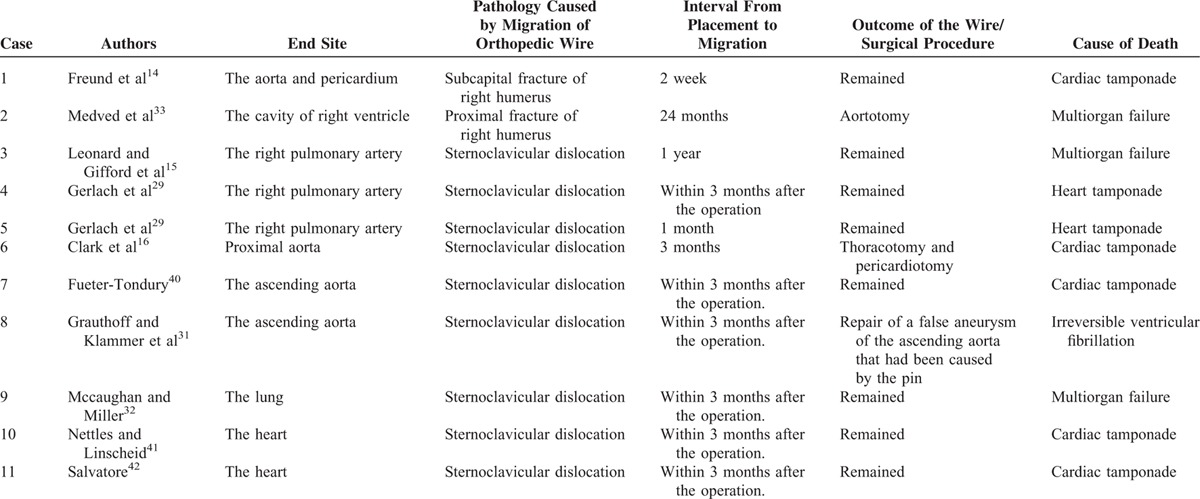
Fatalities From Migration of Orthopedic Wires: Review of the Literature

In our report, the K-wire was used for the fixation of the fracture of the right clavicle and its migration had resulted in perforation of the ascending aorta. Our patient developed syncope, chest pain, and shortness of breath. Though there was no hemothorax or hemomediastinum, the patient developed hemopericardium and cardiac tamponade. Once intra-aortic migration of a K-wire is recognized by CT scan imaging, urgent removal is mandatory due to high risk of fatality related to catastrophic cardiovascular events. Our patient suffered cardiac arrest during induction of intravenous anesthesia and endotracheal intubation. The cardiac arrest may have occurred due to the stimulation of the larynx and trachea during endotracheal intubation, which may have, in turn, stimulated the sympathetic nerve, leading to increased heart rate and oxygen consumption of cardiac muscle. As the peripheral resistance increased due to the acute pericardial tamponade, the burden on the heart increased, leading to myocardial ischemia, eventually causing cardiac arrest.

The exact cause and mechanism of wire migration is obscure. Various causative mechanisms have been proposed, including muscular activity, movement of the shoulder, negative intrathoracic pressure during respiratory excursion, regional resorption of the bone, gravitational force, and even capillary action.^34,36^ The short-term migration of K-wire in unstable osteosynthesis may occur due to the large range of motion of the shoulder, exacerbated by respiratory motion, intrathoracic depression and gravity.^43^ The short time interval between implant and migration of the wire in the present case may be either related to technique (inadequate engagement of the orthopedic wire in the bone cortex), or may be related to factors, such as respiratory excursion, physical activity or gravitational forces.^44^ The long-term migration seems to be due to muscle movement, inducing migration along the arteriovenous circuit in contact with the muscles, and under the effect of gravity.^2^ The K-wires may also rupture and migrate through the circulatory system mortality.^18,45,46,49^

The present report draws attention to the risk of mortality after intraaortic migration of K-wires. All kinds of wires (smooth, threaded, or bent) have been reported to migrate.^47^ Hence, their use should be restricted as much as possible, especially in shoulder surgery. Certain studies have even suggested that K-wire osteosynthesis should be contraindicated.^36^

Given below are a few recommendations regarding the use of orthopedic wires in the shoulder region.

1. Patients managed by K-wire should be informed of the risks associated with it.
2. The number of K-wires implanted during the surgery should be specified in the surgical report.
3. The wire should be curved at its extremity.
4. The patients should be followed regularly, both clinically and radiographically, until removal.
5. Removal of the K-wire should be performed as soon as the treatment period is over.
6. Migrated wires should be removed immediately.

## CONCLUSION

As the migration of wires and pins during orthopedic surgery can cause potentially fatal complications, these should be used very cautiously, especially for percutaneous treatment of shoulder girdle fractures. The patients with such implants should be followed frequently, both clinically and radiographically. If migration occurs, the patient should be closely monitored for emergent complications and the K-wire should be extracted immediately.
